# MALDI Mass Spectrometry Imaging Reveals the Existence of an *N*-Acyl-homoserine Lactone Quorum Sensing System in *Pseudomonas putida* Biofilms

**DOI:** 10.3390/metabo12111148

**Published:** 2022-11-21

**Authors:** Rattanaburi Pitchapa, Sivamoke Dissook, Sastia Prama Putri, Eiichiro Fukusaki, Shuichi Shimma

**Affiliations:** 1 Department of Biotechnology, Graduate School of Engineering, Osaka University, 2-1 Yamadaoka, Suita 5650871, Osaka, Japan; 2 Osaka University Shimadzu Analytical Innovation Laboratory, Osaka University, 2-1 Yamadaoka, Suita 5650871, Osaka, Japan

**Keywords:** mass spectrometry imaging, quorum sensing, *Pseudomonas putida*, biofilms, sample preparations

## Abstract

Quorum sensing (QS) is generally used to describe the process involving the release and recognition of signaling molecules, such as *N*-acyl-homoserine lactones, by bacteria to coordinate their response to population density and biofilm development. However, detailed information on the heterogeneity of QS metabolites in biofilms remains largely unknown. Here, we describe the utilization of matrix-assisted laser desorption/ionization (MALDI) mass spectrometry imaging (MSI) to follow the production of specific metabolites, including QS metabolites, during *Pseudomonas putida* biofilm development. To do so, a method to grow an agar-based biofilm was first established, and MALDI-MSI was used to detect and visualize the distribution of QS metabolites in biofilms at different cultivation times. This study demonstrated that *N*-acyl-homoserine lactones are homogeneously produced in the early stages of *P. putida* biofilm formation. In contrast, the spatial distribution of quinolones and pyochelin correlated with the swarming motility of *P. putida* in mature biofilms. These two metabolites are involved in the production of extracellular polymeric substances and iron chelators. Our study thus contributes to establishing the specific temporal regulation and spatial distribution of *N*-acyl-homoserine lactone-related metabolites and quinolone and pyochelin in *P. putida* biofilms.

## 1. Introduction

Biofilms are multicellular microbial communities that originate from single or multiple microbial species [1]. The biofilm structure acts as a barrier to protect encased microorganisms from environmental and biological hazards. These microbial communities can survive in harsh environments, such as on the inside of oil pipelines and at the liquid–air interface in hydrocarbon fuel tanks [2]. Notably, even in single-species biofilms, subpopulations of cells present at different locations in the structure show distinct expression patterns and secrete different metabolites [3]. Quorum sensing (QS) is a cell–cell communication mechanism involving increasing cell density at the group level, controlled by chemical signal molecules [4,5]. *N*-acyl-homoserine lactones (AHLs) have been extensively studied among the various QS molecules identified. AHLs have been shown to control the expression of numerous traits, including bioluminescence, virulence, symbiosis, different forms of motility, biofilm formation, production of antibiotics and toxins, conjugation, and metal chelation [6,7,8].

Biofilm formation is generally surface-specific. Therefore, research concerning the biofilms’ chemical structure, temporal regulation, and spatial distribution is necessary to understand how they have formed and the direct consequences of their presence. Moreover, understanding the role of QS in biofilm architecture is necessary to treat or prevent problems arising from microbial biofilm formation. *Pseudomonas* is a commonly used model microorganism for studying biofilm formation, with applications in industrial and clinical fields. [9]. Their biofilms are controlled by the AHL-QS system, similar to that observed in a large number of gram-negative bacterial species [10,11].

*Pseudomonas putida* is a well-studied biofilm-forming bacterium known to promote microbiologically influenced corrosion, a process threatening the integrity of oil and sewage pipelines, historic buildings, and military assets [12]. However, knowledge of the *P. putida* QS system and its role in biofilm formation is limited, even though mass spectrometry imaging (MSI) has recently been used as a promising technique to image quinolones, surfactants, and antibiotics in biofilms [13,14]. In contrast to other existing chemical imaging technologies, MSI does not require any molecular labeling mechanism and can be used to identify multiple molecular species in a single experiment [15]. This technique employs a matrix homogeneously applied to sectioned samples to facilitate the desorption/ionization of compounds and reduce fragmentation during laser irradiation. Mass spectral data were collected and collated to generate images of ions whose intensity is related to their abundance. Ion images reveal the spatial distribution of metabolites within the sample [16,17]. In this study, we employed a combination of MSI and matrix-assisted laser desorption/ionization (MALDI) to visualize the heterogeneity of QS metabolites in *P. putida* biofilms. Although MSI is frequently employed in the study of microbial biofilms, several challenges linked to the hydrated, absorbent, deformable, soft, and non-uniform nature of the biofilm surface remain in obtaining reliable ion images [18]. Herein, we focus on developing a cultivation and sample preparation method for widely targeted MS-based chemical imaging of agar-based biofilms.

## 2. Material and Methods

### 2.1. Bacterial Strain and Reagents

*P. putida* 6157 (RIKEN, Saitama, Japan) was used for biofilm cultivation. All chemicals used for the LB media were purchased from Becton Dickinson (Sparks, MD, USA). The MALDI matrices, 2,5-dihydroxybenzoic acid (DHB), and α-cyano-4-hydroxycinnamic acid (CHCA), were purchased from Sigma-Aldrich (St. Louis, MO, USA). Methanol was purchased from WAKO Pure Chemical Industries (Osaka, Japan). Ultrapure water was obtained using a Genpure UV-TOC xCAD Plus (Thermo Fisher Scientific, Waltham, MA, USA).

### 2.2. Process of Agar-Based Biofilm Cultivation

*P. putida* 6157 was first cultured at 26 °C for 15 h in the stationary phase. (OD_600_ = 0.05, ~1.0 × 10^6^ CFU/mL) in LB liquid medium from a single colony. The culture (1 μL) was subsequently inoculated on thin layer LB agar medium (10 mL in a standard 10-cm petri dish, and typically contained 2% *w*/*v* agar), which contained the indium tin oxide (ITO)-coated glass slide for MALDI-MSI (100 Ω/m^2^ without anti-peeling coating, Matsunami Glass, Osaka, Japan). The Petri dishes were sealed with Parafilm to minimize dehydration of the thin agar during incubation. The biofilm was subsequently cultivated under static conditions at 26 °C for 48 h to obtain mature biofilms. For the time-course analysis, agar-based biofilms were sampled at 6, 12, 24, and 48 h to examine the distribution of QS metabolites within biofilms associated with the different stages of biofilm formation.

### 2.3. MALDI Matrix Application

The MALDI matrices DHB and CHCA were applied to the biofilm surfaces via sublimation. Spraying using manual airbrush application was also explored for initial experiments; however, this application method resulted in low detectability of QS signals in biofilms. Therefore, sublimation was selected for the subsequent experiments. The thickness of the DHB matrix was optimized at 0.5 µm for coverage homogeneity and sensitivity. For sublimation, DHB was heated at 180 °C and deposited onto the surface of biofilms in an iMLayer (Shimadzu, Kyoto, Japan).

### 2.4. MALDI-MSI Analysis

MSI was performed using an iMScope TRIO instrument (Shimadzu). Both optical images under microscopic conditions and ion distribution images could be obtained using the same instrument under atmospheric pressure. An Nd:YAG laser (λ = 355 nm, 1 kHz) was used as the MALDI laser source, and laser irradiation was repeated 100 times on each pixel with a laser power of 45.0 (in arbitrary units). Mass spectra were acquired in the positive-ion detection mode with an m/z range of 100–1000 for visualization of QS metabolites in *P. putida* 6157 biofilms. After obtaining the mass spectra, the peak intensity maps were reconstructed using Imaging MS Solution software (Shimadzu, Japan).

### 2.5. Metabolite Annotation

MS/MS analysis with a collision energy of 20 or 40 (arbitrary unit in iMScope TRIO) was performed to confirm the selected precursor *m*/*z* (protonated ion) with predicted QS signals obtained from High Chem Mass Frontier 7.0 (HighChem, Ltd., Bratislava, Slovak Republic), a software package for the management, evaluation, and interpretation of mass spectra. This software provides several useful tools for processing and organizing mass spectral and chromatographic data.

### 2.6. Genome Analysis

Detailed methods are shown in the Supporting Information S1.

## 3. Results

### 3.1. Modified Agar-Based Biofilms for MALDI-MSI

**Figure 1:**
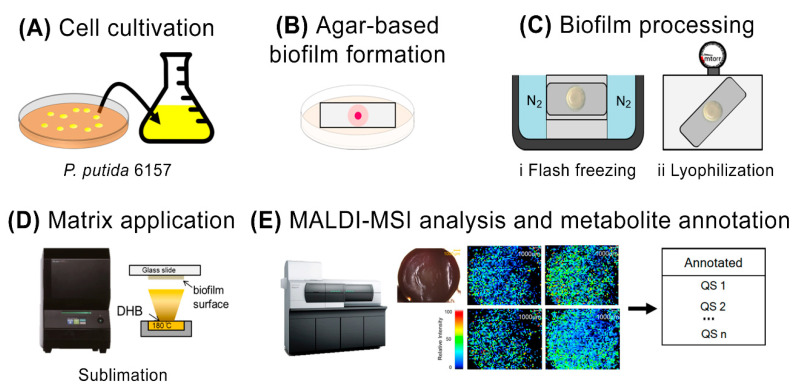
Workflow for cultivation and preservation of agar-based biofilms. (**A**) Cell cultivation. (**B**) Adherence and formation of bacterial biofilms onto the ITO glass slide surface. (**C**) Pretreatment of biofilms by flash-freezing and lyophilization. (**D**) Matrix sublimation and (**E**) MALDI-MSI analysis and metabolite annotation to distinguish ions specific to different biofilm regions.

**Figure 2:**
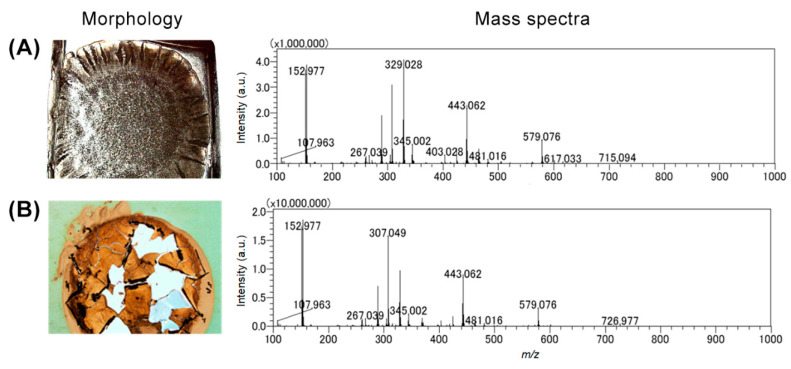
Morphology of modified agar-based biofilms and the mass spectra obtained with (**A**) and without biofilm processing (**B**).

The workflow used for the construction, cultivation, and characterization of the agar-based biofilms for MALDI-MSI is shown in Figure 1. The MALDI-MSI analysis workflow starts with the cultivation of *P. putida* directly on an ITO-coated glass slide embedded in a thin layer of agar medium prior to inoculation with *P. putida*. This device was deposited in the center of Petri dishes (Figure 1A,B) and incubated for various times at 26 °C. After incubation, the biofilm and surrounding agar attached to the glass slide were directly used for MSI analysis (Figure 1C,E and Figure 2). However, the biofilm sample showed insufficient adherence to the target surface [19]. Substantial peeling and flaking of the biofilm were observed during the sublimation process, which involved a change from normal to high vacuum pressure inside the instrument (Figure 2B). The term flaking is used to describe air bubbles, fissures, and partial or complete detachment of the dehydrated biofilm sample from the ITO glass slide. To address this adherence issue and minimize flaking, the biofilm samples were subjected to specific biofilm processing, including flash-freezing in liquid nitrogen and lyophilization (Figure 1C). This step was performed to ensure that good biofilm morphology was preserved during sublimation. Furthermore, we confirmed that the number of detected peaks was the same with and without the biofilm flash-freezing and lyophilization steps (Figure 2A). Two commonly used MALDI matrices, DHB and CHCA, were tested and sublimated onto the surfaces of agar-based biofilm samples. The DHB matrix powder provided a more homogeneous matrix crystal and broader coverage of a higher number of detected metabolites (including AHL-QS molecules) than the CHCA matrix powder (Figure 2 mass spectra). After the DHB matrix was sublimated onto the biofilm surfaces, MALDI-MSI and metabolite annotation were performed (Figure 1D,E).

### 3.2. Correlation between the Production of AHL Metabolites and Biofilm Development in P. putida 6157

**Figure 3:**
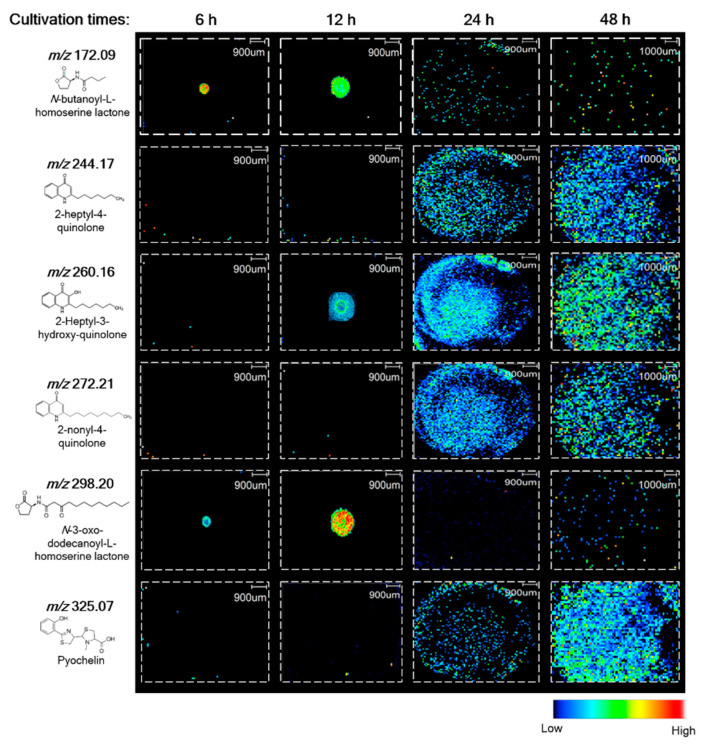
MSI images of *N*-acyl-homoserine lactone quorum sensing metabolites produced by biofilms *P. putida* 6157 grown for 6, 12, 24, and 48 h.

To elucidate the relationship between the spatial distribution of QS metabolites produced by *P. putida* 6157 and the stage of biofilm formation, we first performed MS analysis of the biofilm samples incubated at 26 °C for 6, 12, 24, and 48 h after inoculation. The biofilms were grown for 6 and 12 h represented the early stage of the biofilm, whereas those grown for 24 and 48 h showed features of mature biofilms. The QS metabolites detected and mapped on *P. putida* biofilms were not the same at different cultivation times (Figure 3). Two distinct ion groups, including six *m*/*z*, were observed as QS metabolites in *P. putida* biofilms. The first group included *m*/*z* 172.09 and *m*/*z* 298.20, annotated as *N*-butanoyl-L-homoserine lactone (C4-HSL) and *N*-3-oxo-dodecanoyl-L-homoserine lactone (3-oxo-C12-HSL), respectively. These two metabolites were found only in early-stage biofilms and were primarily localized in the region of inoculation. The second group included QS metabolites that could only be visualized in the later stages of biofilm development. This group was composed of quinolone metabolites, 2-heptyl-4-quinolone (HHQ), 2-heptyl-3-hydroxy-quinolone (PQS), and 2-nonyl-4-quinolone (NHQ) and was homogeneously distributed on the surface of *P. putida* 6157 biofilms. Our experimental *m*/*z* values of annotated AHLs and quinolones for the protonated molecular ion ([M + H]^+^) were similar to the data from previous studies, indicating good validation of our analysis method, as presented in Table 1 [20,21]. The localization abundance pattern of this second group was more compatible with swarming motility, resulting in the mass movement of cells and, thus, mature biofilm formation. Notably, PQS was the only quinolone observed during the early stages of biofilm formation at 12 h. Pyochelin, a siderophore involved in iron chelation, was only visualized in the mature stage of *P. putida* 6157 biofilms, mainly at the biofilm’s front line. In contrast, the repartitioning of the other quinolones was homogenous in the biofilm. These results indicated a clear relationship between the temporal production and spatial distribution of QS metabolites and biofilm development in *P. putida* 6157.

## 4. Discussion

The development of the method for agar-based biofilm formation described in this study takes several factors into consideration, including wide application range, ease of operation, reproducible performance, and reduced risk of contamination. The agar-based biofilm approach proposed in this study was used to prepare biofilm samples for MSI because it has a lower risk of contamination. Furthermore, it can be used to study more than one colony per target plate, increasing sample throughput and allowing numerous samples to be processed via a single MALDI-MSI analysis. Previous studies have reported the visualization of QS molecules in *P. aeruginosa* using MALDI-MSI [22,23]. Several reports have suggested that the same QS metabolites detected in *P. aeruginosa* also exist in some *P. putida* strains, such as KT2440 [24] and IsoF [25]. However, before this report, the visualization of QS metabolites in *P. putida* via MALDI-MSI had not been successful, and this may be due to the difficulties associated with sample preparation. Recently, Li et al. [26] demonstrated the use of a low-shear drip flow reactor for biofilm formation in *P. putida* F1. Using this approach, robust biofilms for MSI analysis were obtained within 72 h of inoculation. The spatial distribution of lipids and oligosaccharides was reported in this study; however, attempts to visualize QS metabolites were unsuccessful. It was reported that the abundance of QS compounds dramatically decreased in 48 h biofilms [27]. Therefore, a new method for the preparation of robust 48 h biofilms is required for MALDI-MSI analysis of QS metabolites and their distribution in *P. putida* biofilms.

**Figure 4:**
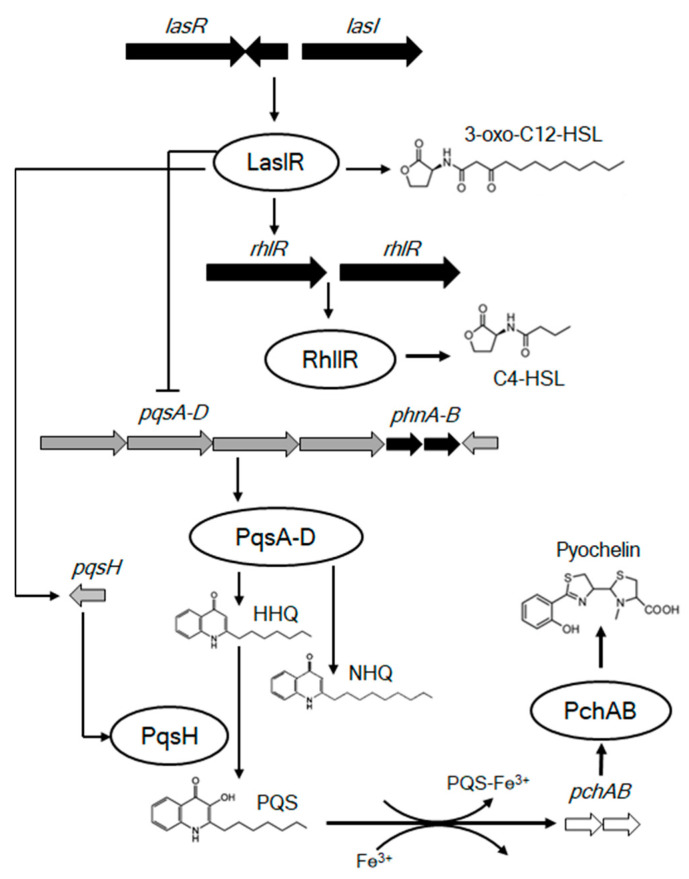
The hierarchical *N*-acyl-homoserine lactone quorum sensing system in *P. putida* 6157 biofilm. LasI-LasR contains genes that produced 3-oxo-C12-HSL. While the RhlI-RhlR system generates C4-HSL. AHL-QS are linked as LasR/3-oxo-C12-HSL is required for full expression of *pqsH*, which controls the production of PQS. The *pqs* group is repressed by the action of the RhlR/C4-HSL system. HHQ and NHQ are produced via PqsA-D system itself. Furthermore, PQS released from the cell is capable of binding iron (PQS-Fe^3+^ complex). The removal of iron by PQS induces expression of genes involved in siderophore production, leading to pyochelin production.

Our modified agar-based biofilm workflow (Figure 1) was the first successful strategy to yield robust and reproducible flat biofilms 48 h after inoculation. These biofilms were used to visualize the heterogeneity of QS metabolites and the correlation between temporal and spatial QS metabolite production and biofilm development in *P. putida*, using MALDI-MSI. Our results showed that C4-HSL and 3-oxo-C12-HSL production was only observed in young biofilms. This agrees with previous work on biofilm formation and QS metabolite production using liquid chromatography coupled with mass spectrometry (LC-MS) [28]. In addition, we demonstrated that the production and distribution of AHLs are correlated with swarming mortality, which is crucial for the formation of mature biofilm structures. Quinolone and pyochelin distribution were visualized for the first time in *P. putida,* and our results demonstrated that these metabolites were only observed in mature biofilms. Previous reports have shown that the AHL-QS system is controlled by a transcriptional factor pair, RhlI-RhlR, subordinate to the LasI-LasR pair. LasI is involved in the production of 3-oxoC12-HSL and RhlI in C4-HSL, both of which are produced at the early stage of biofilm formation. These AHLs bind to the transcriptional activators LasR and RhlR and activate target promoters. Both *las* and *rhl* have been implicated in the regulation of quinolone-like signaling molecule production in a group of *Pseudomonas* [29,30]. As C4-HSL and 3-oxo-C12-HSL are quinolone precursors, they could not be detected when quinolones were highly homogenously produced throughout the *P. putida* biofilm. AHLs and quinolones have been reported to regulate the expression of genes required for specialized metabolite production, including that of the siderophore, pyochelin [31]. This is consistent with our data, which demonstrated that pyochelin was observed only in mature biofilms using PQS as a precursor (Figure 4).

Our study demonstrated that QS heterogeneity could be visualized using a modified agar-based biofilm approach, which is convenient for MALDI-MSI analysis. The workflow used in our study was simple and could easily be adapted to various microbial biofilms. This study revealed the relationship between biofilm development and spatial production of specific QS metabolites in *P. putida*. These findings expand our knowledge of QS and the mechanisms of AHL-QS-based biofilm formation. Furthermore, the QS metabolites detected in our study could be used as targets to impair the development of pathogen biofilms during infection.

## Figures and Tables

**Table 1:** *N*-acyl-homoserine lactones and quinolones observed in *P. putida* 6157 biofilms.

Compound ID	Molecular Formula	[M + H]^+^ Theoretical	[M + H]^+^ Exp.	References
C4-HSL	C_8_H_13_NO_3_	172.09	172.09	[20]
3-oxo-C12-HSL	C_16_H_27_NO_4_	298.20	298.20	[20]
HHQ	C_16_H_21_NO	244.17	244.17	[21]
PQS	C_16_H_21_NO_2_	260.17	260.16	[21]
NHQ	C_18_H_25_NO	272.21	272.21	[21]
Pyochelin	C_14_H_16_N_2_O_3_S_2_	325.06	325.07	This work

Abbreviations: C4-HSL, *N*-butanoyl-L-homoserine lactone; 3-oxo-C12-HSL, *N*-3-oxo-dodecanoyl-L-homoserine lactone; HHQ, 2-heptyl-4-quinolone; PQS, 2-heptyl-3-hydroxy-quinolone; NHQ, 2-nonyl-4-quinolone.

## Data Availability

The data presented in this study are available in the article and supplementary material.

## Supplementary Materials

The following supporting information can be downloaded at: https://www.mdpi.com/article/10.3390/metabo12111148/s1, Information S1: DNA sequence analysis of *P. putida* 6157.
