# Supplementary material for: MALDI Mass Spectrometry Imaging Reveals the Existence of an N-Acyl-homoserine Lactone Quorum Sensing System in Pseudomonas putida Biofilms
Source: Metabolites. 2022 Nov 21;12(11):1148. doi: 10.3390/metabo12111148 (PMC9697013; doi:10.3390/metabo12111148)
Supplement: Supplementary file 1 [file metabolites-12-01148-s001.zip › metabolites-2011005-supplementary.pdf]

Supportive information S1

DNA sequence analysis of *P. putida* 6157

Whole-genome sequencing was conducted with the illumina NovaSeq 6000 sequencing platform on genomic DNA isolated from *P. putida* 6157 to confirm the existence of the AHLs-QS system. Sequencing is done using a 150-bp paired-end sequencing technique generated from 1-kbp mate-pair libraries, in which the raw reads were assessed with Fasqc software, and the low-quality reads were removed using the default parameter of the Trimmomatic software. High-quality reads were subsequently assembled using SPAdes software, and the gene was called using Prokka software. The potential gene was then aligned with the reference genome sequence of *P. aeruginosa* using the Basic Local Alignment Search Tool (BLAST, NCBI) for gene annotation. Protein sequence alignments could predict if the proteins have a similar function when the pairwise sequence identity is >25% for long alignments. The presence of lasIR, rhlIR, and PQS clusters in the genome of *P. putida* was firstly reported by this study, leading to the production of C4-HSL, 3-oxo-C12-HSL and PQS visualized by MALDI-MSI. Sequences producing significant alignments obtained from this work consisted of QS regulator LasR (53%), QS regulator RhlR (52%), anthranilate synthase component I (98%), anthranilate synthase component II (97%), anthranilate-CoA ligase (28%), 2-heptyl-4(1H)-quinolone synthase subunit PqsC (24%), anthraniloyl-CoA anthraniloyltransferase (29%), 2-heptyl-3-hydroxy-4(1H)-quinolone synthase (26%), QS regulator MvfR (30%), rhamnosyltransferase subunit A (93%), salicylate biosynthesis isochorismate synthase (29%), and isochorismate pyruvate lyase (31%)
